# The value of the “Surgical Risk Preoperative Assessment System” (SURPAS) in preoperative consultation for elective surgery: a pilot study

**DOI:** 10.1186/s13037-020-00256-4

**Published:** 2020-07-25

**Authors:** Michael R. Bronsert, Anne Lambert-Kerzner, William G. Henderson, Karl E. Hammermeister, Chisom Atuanya, Davis M. Aasen, Abhinav B. Singh, Robert A. Meguid

**Affiliations:** 1 Surgical Outcomes and Applied Research, University of Colorado School of Medicine, Aurora, CO USA; 2 Adult and Child Consortium for Health Outcomes Research and Delivery Science, University of Colorado School of Medicine, Aurora, CO USA; 3 Department of Health Systems, Management, and Policy, Colorado School of Public Health, University of Colorado School of Medicine, Aurora, CO USA; 4 Department of Biostatistics and Informatics, Colorado School of Public Health, Aurora, CO USA; 5 Division of Cardiology, University of Colorado School of Medicine, Aurora, CO USA; 6 Division of Cardiothoracic Surgery, Department of Surgery, University of Colorado School of Medicine, University of Colorado, Denver Anschutz Medical Campus, 12631 E. 17th Avenue, C-310, Aurora, CO 80045 USA

**Keywords:** SURPAS, Surgical risk prediction, Mixed methods

## Abstract

**Background:**

Risk assessment is essential to informed decision making in surgery. Preoperative use of the Surgical Risk Preoperative Assessment System (SURPAS) providing individualized risk assessment, may enhance informed consent. We assessed patient and provider perceptions of SURPAS as a risk assessment tool.

**Methods:**

A convergent mixed-methods study assessed SURPAS’s trial implementation, concurrently collecting quantitative and qualitative data, separately analyzing it, and integrating the results. Patients and providers were surveyed and interviewed on their opinion of how SURPAS impacted the preoperative encounter. Relationships between patient risk and patient and provider assessment of SURPAS were examined.

**Results:**

A total of 197 patients were provided their SURPAS postoperative risk estimates in nine surgeon’s clinics. Of the total patients, 98.8% reported they understood their surgical risks very or quite well after exposure to SURPAS; 92.7% reported SURPAS was very helpful or helpful. Providers shared that 83.4% of the time they reported SURPAS was very or somewhat helpful; 44.7% of the time the providers reported it changed their interaction with the patient and this change was beneficial 94.3% of the time. As patient risk increased, providers reported that SURPAS was increasingly helpful (*p* < 0.0001).

**Conclusions:**

Patients and providers reported the use of SURPAS helpful and informative during the preoperative risk assessment of patients, thus improving the surgical decision making process. Patients thought that SURPAS was helpful regardless of their risk level, whereas providers thought that SURPAS was more helpful in higher risk patients.

## Background

The rates of perioperative mortality and morbidity following surgery remain of great concern. The occurrence of a perioperative complication has been shown to reduce patients’ quality of life [1–3], longevity [4, 5], and substantially increase costs [6, 7]. Therefore, providing accurate pre-surgical risk assessment to patients is essential to support an informed decision regarding surgery [8–10] and increases the burden on surgeons to provide more complete and interpretable information about any operation [9, 10].

Currently, risk assessment in surgery is variable in practice [11, 12] and is typically based on accepted or previously reported statistics, and subjective assessment of individual patient comorbidities by providers [9, 13]. Formal risk assessment tools exist, such as the American College of Surgeons’ (ACS) Surgical Risk Calculator and the Veterans Affairs Surgical Quality Improvement Program (VASQIP) Risk Calculator. Unfortunately, these and other tools require considerable time to use and are not integrated into clinical workflow or electronic health records (EHR) [13]. Additionally, the ACS Surgical Risk Calculator has limitations regarding accuracy of risk estimates for higher risk patients. These issues are addressed by the Surgical Risk Preoperative Assessment System (SURPAS) [14–17].

SURPAS is user-friendly and integrated into the EHR. SURPAS is based on a set of eight risk variables that are readily available at the preoperative visit and provides accurate risk estimates of 12 significant adverse surgical outcomes across nine adult surgical specialties. SURPAS was developed from the American College of Surgeons National Surgical Quality Improvement Program (ACS NSQIP) database and the design and statistical methodologies of SURPAS have been described elsewhere [14–19]. The input data and estimated surgical risks can be incorporated into the patient’s permanent medical record in providers’ notes. Additionally, a pictorial and numeric display of the results are printed out and provided to the patient for subsequent reference. Therefore, SURPAS has the potential to impact an informed decision making by improving patient communication and health literacy, in addition to helping inform provider understanding of individual patient perioperative needs and care [20]. We assessed patient and provider perceptions of SURPAS as a risk assessment tool during a trial implementation.

## Methods

We initiated a trial implementation of SURPAS in elective surgical patients across a broad range of surgical clinics. We evaluated this trial implementation assessing patient and provider perceptions of SURPAS as a risk assessment tool utilizing a mixed methodology to assess the utilization of SURPAS. Mixed methods are used when combining qualitative and quantitative research methods during the research process to provide more informative and useful results [19]. A convergent mixed-methods design collects both qualitative and quantitative data concurrently and merges the two forms of data to address the study aims [19]. After data analysis, the quantitative and qualitative data are compared, contrasted and interpreted [19]. In this study, we collected quantitative surveys of each patient and surgical provider after each encounter. We also interviewed a sample of the patients and all the providers to assess their opinions of SURPAS. The findings from the trial implementation, reported in this manuscript, will be used to design and implement a SURPAS broad dissemination and implementation protocol. This study was approved by the Colorado Multiple Institutional Review Board (# 15–1044).

### Participants and setting

Recruitment for the trial implementation included surgical providers from the University of Colorado School of Medicine Department of Surgery and patients seen in their outpatient surgical clinics at the University of Colorado Hospital. The surgical providers who agreed to participate were provided post-card consents. The providers were asked to use the SURPAS tool with their patients during the consent process, complete a quantitative survey on it after use with each patient, and agree to be interviewed. Surgical patients of the recruited surgeons were consented and provided a copy of the signed informed consent. Their participation included utilization of SURPAS during their risk discussion, completion of a quantitative survey, and a possible interview to obtain their opinions of the experience with SURPAS.

### Data collection and analyses

#### Qualitative inquiry

The qualitative inquiry obtained patient and provider opinions on, and any suggestions to improve SURPAS. It solicited thoughts on the local culture and its proclivity for change, the implementation climate of the clinical environment, and suggestions to optimize the implementation process to utilize the SURPAS tool. We interviewed providers (surgeons and nurse practitioners) who implemented SURPAS and a convenience sampling of patients who experienced SURPAS utilizing specific interview guides (eAttachments A and B) during their pre-surgical risk assessment to obtain their opinions of the tool and how it was used in their clinical encounter. The sample of patients who were interviewed were selected during a one-week period where the interviewer (ALK) was present in the individual clinics and interviewed the recruited patients in the time period. The data were assessed to ensure saturation (meaning no new information was being shared). The 30–45 min interviews were audiotaped and transcribed verbatim.

#### Qualitative analyses

An inductive and deductive toolkit of analytical strategies, drawing primarily on matrix and reflexive analysis, was used to analyze the qualitative data [21, 22]. Defined segments of transcribed text were coded to create categories and themes. A matrix analysis was created using a priori codes and novel codes which emerged from participant responses. The validity and accuracy/reliability of the early codes were established by the qualitative methodologist (ALK), who analyzed the initial transcripts and defined the initial codebook [21–24]. Subsequent transcripts were analyzed, and emergent codes were added throughout analysis. Analysis of the codes resulted in the emergence of themes. The consistency of coding/interpretation was reviewed by all co-authors at regular group meetings and discrepancies were addressed through discussion and consensus. This process continued until thematic saturation was achieved, meaning no new themes emerged. All analyses and findings were integrated and documented with an audit trail [21–24]. Participant quotes were selected by consensus of all members of the analytic team to ensure representativeness across interviews.

#### Quantitative inquiry

Two forms of quantitative data were collected: 1) The consented providers and patients were provided a paper self-administered survey at their pre-surgical visit, and 2) the patient’s risk estimates for mortality, morbidity, and unplanned readmission were calculated by SURPAS. The SURPAS data were merged with the patient and provider survey data for analysis.

#### Quantitative analysis

We calculated descriptive statistics of the study population using proportions and means for patient demographics and predictors used to calculate individual patient risk estimates. For both patient and provider surveys we calculated the frequency distribution for each item. To evaluate if there were any relationships between patient risk estimates and patient and provider responses to the four survey questions specifically asking about the SURPAS tool, we compared the median risk values for mortality, morbidity, and unplanned readmission for the different levels of each survey question. We used the Wilcoxon rank-sum test since the risk estimates were skewed; this is a nonparametric test that does not require the assumption of a normal distribution.

All statistical tests were considered to be significant at a two-sided *P* < 0.05. All analyses were performed using SAS software version 9.4 (SAS Inc., Cary, NC).

## Results

### Demographic characteristics of the patient sample

A total of 197 patients were enrolled during preoperative consultation. Their mean age was 54.8 (Standard Deviation (SD) 16.3), with 54.8% of the patients being female, 83.8% Caucasian, 5.6% African American, and 8.1% Hispanic (Table 1). This patient population compared well with the overall demographics of patients being seen in preoperative clinics at the institution in the time period of the study, January 1 to July 31, 2017. The mean age of 52.4 years (SD 16.7), with 55.8% female patients, 69.8% Caucasian, 8.0% African American, and 12.3% Hispanic (eTable 1). The convenience sample of patients interviewed (*n* = 27) had similar characteristics to the 197 patients who were surveyed: mean age 55.5 (SD [13.6]); 55.6% female; 100% Caucasian; and 7.4% Hispanic.

**Table 1:** Patient’s survey and interview demographics

Demographics^a^	Patient Cohort (*n* = 197) N (%)	Not Interviewed Cohort (*n* = 170) N (%)	Interviewed Cohort (*n* = 27) N (%)
Age, years, mean (SD)	54.8 (16.3)	54.7 (16.7)	55.5 (13.6)
Gender			
Female	108 (54.8)	93 (54.7)	15 (55.6)
Male	89 (45.2)	77 (45.3)	12 (44.4)
Race			
Caucasian	165 (83.8)	138 (81.2)	27 (100)
Black or African American	11 (5.6)	11 (6.5)	0 (0)
Asian	6 (3.1)	6 (3.5)	0 (0)
American Indian or Alaskan Native	1 (0.5)	1 (0.6)	0 (0)
Missing racial information	14 (7.1)	14 (8.2)	0 (0)
Hispanic Ethnicity			
No	176 (89.3)	152 (89.4)	24 (88.9)
Yes	16 (8.1)	14 (8.2)	2 (7.4)
Unknown	5 (2.5)	4 (2.4)	1 (3.7)

^a^*Abbreviation SD* standard deviation

### Descriptive statistics of the SURPAS risk predictors of the patient sample

Of the 197 patients entered into the study, 180 (91.4%) had the SURPAS evaluation. Mean age was 54.0 (SD [16.6]), 93.9% had independent functional status and 51.1% were undergoing outpatient surgery. In addition, eTable 2 lists the procedures that patients underwent. The majority of patients were American Society of Anesthesiology physical status classification (ASA class) of III or less (92.2%). Most of the patients were seen by a general surgeon (48.9%), thoracic surgeon (28.3%), or vascular surgeon (9.4%). Only one patient surgery was undergoing an emergency operation. Average work RVU was 15.2 (SD [8.6]). The median SURPAS risk estimates for 30-day mortality, overall morbidity, and unplanned readmission were 0.1, 3.9, and 3.1%, respectively (Supplemental Table 1). This sample of 180 patients was slightly younger, had more outpatient surgeries, had lower ASA class and less emergency operations than the ACS NSQIP national sample during the same time period (Supplemental Table 2).

Of the 17 patients who did not have the SURPAS evaluation, eight had missing or incomplete data entered into SURPAS, six did not undergo surgery, and three underwent operations that did not have CPT codes available in SURPAS.

### Demographic characteristics of the provider sample

The nine surgical providers recruited included seven surgeons and two nurse practitioners (NPs) who agreed to participate in the study by implementing SURPAS. The providers who participated in the study included 55.6% females; 55.6% with a MD only; 22.2% with a MD and Master’s degree; and 22.2% who had Masters of Nursing degrees.

### Integrated qualitative and quantitative results

#### Patient surveys and illustrative quotes from patient interviews

Of the 197 patients in this study, 168 (85.3%) patients reported they discussed the risk of their upcoming surgery at their preoperative visit, five patients reported they did not have the risk discussion and four had missing data. The NPs in the pre-procedure clinic do not have risk assessment conversations with the patients, therefore, the 20 patients who were seen by the NPs subsequently did not have a risk discussion or a survey. The SURPAS risk estimates were documented in the patients’ medical record notes for the surgeons to review.

Of those who reported having the risk discussion, the majority of the patients (96.4%) reported that the surgeon used the SURPAS tool during their discussion; 88.1% of them reported that they understood their surgical risk,” very well, I have no further questions’ and 10.7% reporting, “quite well, but I still have a question” (Table 2).

“*They come in and they talk in terms above your head, and they say, "Hope you understood and then they leave. He doesn't. He makes sure you understand." He often asked, "Do you have any questions?" so that was good"* (T4/4)

Of the patients who reported having a risk discussion with their providers, 165 (98.2%) reported that they received the SURPAS hand out that visually outlined their individual risk estimates, with 154 (93.3%) reporting that the SURPAS tool was explained to them; 26 (15.8%), reported that seeing their risk estimates made them want to talk to their providers more and that it affected their decision to have or not have the operation (Table 2).

*“. . . When I came here, I was a lot more worried, even if he said, “Okay, you can get the surgery. We should get the surgery.” But now seeing the percentages and how low they are, I mean, I have faith that it’s gonna work”* (T2/2)

*“I understood it perfectly. The concept is just knowing what risks in each category there was.”*(E75/158)

**Table 2:** Patient’s and provider’s responses to the SURPAS survey questions

Patient Survey Questions	(*N* = 197) N (%)
Was the risk of your upcoming surgery discussed with you at your preoperative visit today?	
Missing	24 (12.2)
No	5 (2.5)
Yes	168 (85.3)
	(*N* = 168)
Did the surgeon who will do your operation tell you about the risk?	
Missing	1 (0.6)
No	1 (0.6)
Yes	166 (98.8)
Did the surgeon discuss the SURPAS tool with you?	
Missing	1 (0.6)
No	5 (3.0)
Yes	162 (96.4)
How well do you understand the risk of your surgery?	
Missing	2 (1.2)
Very well, I have no further questions	148 (88.1)
Quite well, but I still have a question	18 (10.7)
Somewhat, but I still have a few questions	0 (0)
Minimally, I still have a lot of questions	0 (0)
Not at all	0 (0)
Were you given the papers that show you the estimates of the risks of surgery?	
No	3 (1.8)
Yes	165 (98.2)
	(*N* = 165)
If yes: was this document explained to you?	
Missing	11 (6.7)
No	0 (0)
Yes	154 (93.3)
Was the surgical risk provided by SUPRAS less, the same, greater than you expected?	
Missing	28 (17.0)
Greater than you expected it to be	21 (12.7)
The same	73 (44.2)
Less	43 (26.1)
Did seeing the risk make you want to talk to the provider more about your surgery?	
Missing	21 (12.7)
No	118 (71.5)
Yes	26 (15.8)
Did using the SURPAS tool and seeing the personal risks affect your decision to have or not have the operation?	
Missing	3 (1.8)
No	136 (82.4)
Yes	26 (15.8)
On a scale of 1–4, please circle the number that best reflects your opinion of the risk document given you.	
Missing	4 (2.4)
It was very helpful	100 (60.6)
It was helpful	53 (32.1)
It was somewhat helpful	8 (4.9)
It was not helpful	0 (0)
**Provider’s Responses to the SURPAS Survey**	
Provider’s Survey Questions	(*N* = 197) N (%)
Did you discuss the SURPAS tool with your patients?	
Missing	8 (4.1)
No	23 (11.7)
Yes	166 (84.3)
Did you give the SURPAS handout to the patient?	
Missing	12 (6.1)
No	20 (10.1)
Yes	165 (83.8)
If yes: Did you explain the SURPAS patient handout to the patient?	
Missing	1 (0.6)
No	0 (0)
Yes	164 (99.4)
Was the surgical risk provided from SURPAS	
Missing	32 (16.2)
Less	27 (13.7)
The same	101 (51.3)
Greater than you expected it to be	37 (18.8)
On a scale of 1–4, please circle the number that best reflects your opinion of the risk document given to the patient?	
Missing	14 (7.1)
It was not helpful	19 (9.6)
It was somewhat helpful	46 (23.4)
It was helpful	98 (49.8)
It was very helpful	20 (10.2)
Did using the SURPAS tool change the interaction with the patient during the preoperative clinic visit?	
Missing	14 (7.1)
No	95 (48.2)
Yes	88 (44.7)
If yes: Did you find the change to be:	
Missing	1 (1.1)
Detrimental	0 (0)
Neutral	4 (4.6)
Beneficial	83 (94.3)
Did using the SURPAS tool and seeing the personal risks affect your decision to do or not do the operation?	
Missing	12 (6.1)
No	182 (92.4)
Yes	3 (1.5)
Did using the SURPAS tool and seeing the personal risks change any aspects of the preoperative workup?	
Missing	10 (5.1)
No	178 (90.4)
Yes	9 (4.6)
Did using the SURPAS tool and seeing the personal risks change any aspects of patient management or technical aspects in the operating room?	
Missing	14 (7.1)
No	179 (90.9)
Yes	4 (2.0)
Did using the SURPAS tool and seeing the personal risks change any aspects of the planned postoperative care for this patient?	
Missing	13 (6.6)
No	181 (91.9)
Yes	3 (1.5)

*Abbreviation*: *SURPAS* Surgical Risk Preoperative Assessment System

#### Provider surveys and illustrative quotes from provider interviews

The participating providers completed a survey asking about the patient’s calculated SURPAS risk estimates and their discussion of those risks for each of the 197 patient visits (Table 2). The surgical providers reported that they discussed the SURPAS tool with 166 (84.3%) of their patients (20 patients were seen by the pre-procedure clinic NPs who did not do risk discussions). For these 166 patients, the providers said that they gave the SURPAS handout to 165 patients and explained the handout for 164 patients.

*“I think it’s a great idea and I think it’s super easy to use. I think that to widely implement it and get people to use it, it has to be simple and quick. I have been using it predominantly in my clinic, which is all elective, super-low risk, generally healthy patients, so for my patient population, all of the things (predicted adverse event rates) are less than one percent. No different from the national average or whatever.“* (Provider #3)

*“Very easy to enter the data after you have seen a patient in clinic. Very much like the documentation tool. You can generate the note of what the predicted risks are for that patient compared to the national database and simply copy that into your clinical note.”* (Provider #4)

In 44.7% of the visits the surgeon reported that the SUPRAS tool changed their interaction with the patients, and 94.3% of the time this was ‘beneficial’.

*“You know for the surgeries I do it’s really helpful. Just because it’s a big, long and complicated surgery and it helps patients see that visually. The more ways we can talk around what this is like, the better it is for them. It also can help us in decision making. For some patients that are really that sick, we can choose second line treatment, which may not be quite as good, but if they’re that sick we pick it up from the SURPAS tool.”* (Provider #7)

The providers reported that the SURPAS tool rarely changed the decision to do the operation (1.5%), aspects of the preoperative work-up (4.6%), aspects of patient management or technical aspects in the operating room (2.0%), or aspects of the *postoperative care (1.5%).*

*“Some of the results we've gotten have been surprising to me, in terms of the risks of the surgery for certain patients. I assumed it would be on the higher end of the risk, but the actual number seems higher than what I would've put on the risk... I think it's been helpful for patients to see the numbers, at least from when I talked to them. It feels to me that they're more educated about their operation. It hasn't actually changed what I do, in terms of surgery... I haven't cancelled an operation because of the high risk. I think some of the patients have been reassured by the low risk. Whenever I consent them and I say there's a risk of heart attack, stroke, or death it sounds very bad. When they see that it's 0.8 percent, then I think they feel a little bit better.”* (Provider #10)

*“If SURPAS helps them understand what their risks are in making a decision and understanding how the things that they’re focused on might be better or worse…”* (Provider #4)

#### Comparison of patients and providers to the same questions

**Fig. 1:**
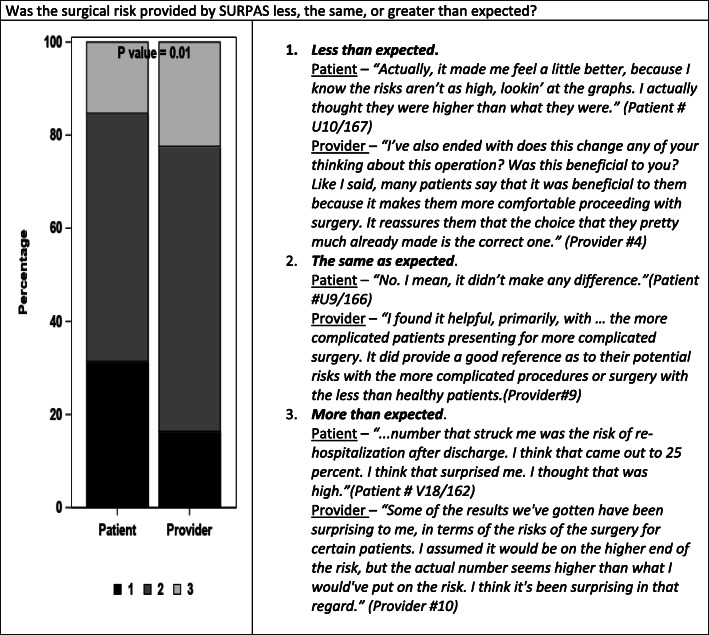
Was the surgical risk provided by SURPAS less, the same, or greater than expected?

**Fig. 2:**
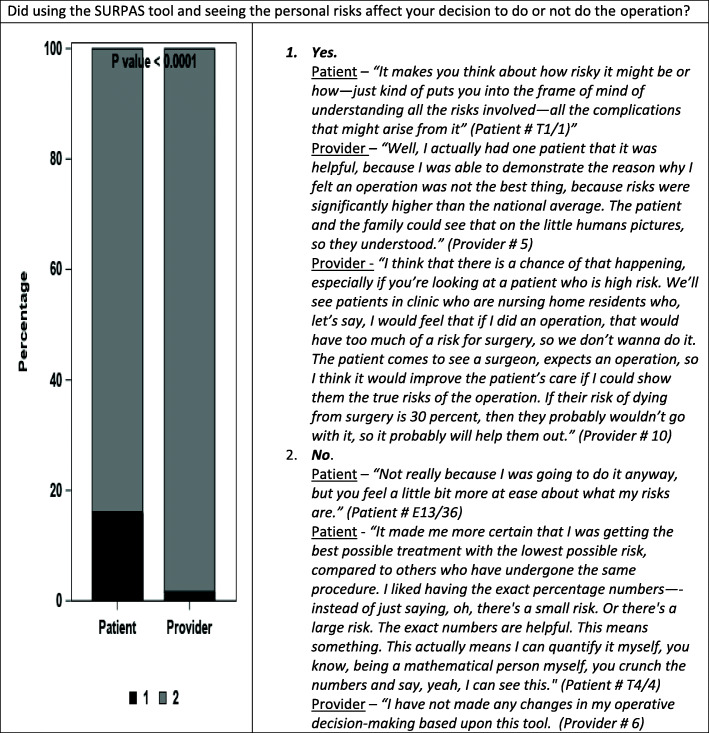
Did using the SURPAS tool and seeing the personal risks affect your decision to do or not do the operation?

**Fig. 3:**
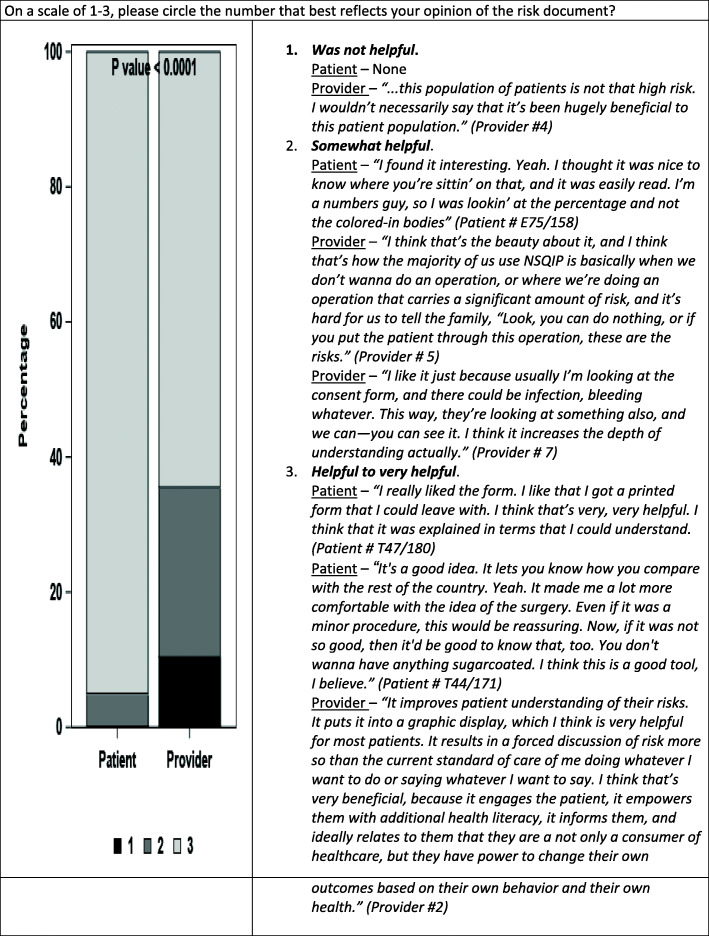
On a scale of 1–3, please circle the number that best reflects your opinion of the risk document?

The left side of Figs. 1, 2, and 3 compare patient and provider answers to three questions that were asked of both patients and providers. The right side of each figure gives illustrative opinions from the individual interviews of the patients and providers.

In response to Question 1) *“Was the surgical risk provided by SURPAS less, the same, or greater than you expected?”* patients tended to overestimate, and surgeons tended to underestimate risk compared to the SURPAS risk estimates (Fig. 1, *p* = 0.01).

In response to Question 2) *“Did using the SURPAS tool and seeing the personal risks affect your decision to [have/do] or not [have/do] the operation?”* a higher percentage of patients (15.7%) compared to providers (1.5%) said that seeing the personal risks affected their decision to have/do or not have/not do the operation (Fig. 2, *p* < 0.0001).

In response to Question 3) “*On a scale of 1-4, please circle the number that best reflects your opinion of the risk document given to you/the patient’ in terms of helpfulness?*” patients rated the risk document as more helpful than did the surgeons (Fig. 3, *p* < 0.0001).

#### Relationship of the patients’ survey answers to the patients’ risk estimates

We obtained the SURPAS calculated risk estimates for mortality, overall morbidity, and unplanned readmission on 180 patients (91.4%) and assessed if there were any relationships between the patient’s SURPAS risk estimates and their responses to the four survey questions specifically asking about the SURPAS tool (Table 3). Patients who responded ‘yes’ to “*Did seeing the risk make you want to talk to the provider more about your surgery?*” tended to have larger median risk estimates for overall morbidity versus patients who responded ‘no’ (4.0% vs. 2.9%: *p* = 0.06). Patient risk was not related to the patient’s perceptions about whether the SURPAS risk was the same, greater, or less than their previous perceptions of their risk, their perceptions about the helpfulness of the risk document, nor their decisions to have or not have the operation.

**Table 3:** Median and interquartile range of risk estimates stratified by response to patient survey question related to the SURPAS tool

Question^a^		30-day mortality		Overall morbidity		Unplanned Readmission	
	N	Median (IQR)	*P* value†	Median (IQR)	*P* value†	Median (IQR)	*P* value†
Was the surgical risk provided from SURPAS:							
The same	70	0.1 (< 0.1–0.3)	0.58	2.7 (1.6–6.6)	0.74	2.3 (1.6–5.1)	0.88
Less	42	0.1 (< 0.1–0.2)		3.5 (1.7–6.5)		2.8 (1.3–4.9)	
Greater than you expected it to be	21	0.1 (< 0.1–0.6)		5.0 (1.5–8.7)		3.2 (1.7–5.9)	
Missing^b^	47						
On a scale of 1–4, please circle the number that best reflects your opinion of the risk document given to you?							
It was not helpful	0	0 (0–0)	0.94	0 (0–0)	0.72	0 (0–0)	0.78
It was somewhat helpful	6	0.1 (< 0.1–1.1)		2.0 (1.1–12.3)		2.0 (1.2–7.5)	
It was helpful	51	0.1 (< 0.1–0.4)		3.3 (1.5–10.5)		2.6 (1.5–5.4)	
It was very helpful	99	0.1 (< 0.1–0.3)		3.5 (1.7–6.9)		3.0 (1.8–5.1)	
Missing^b^	24						
Did seeing the risks make you want to talk to the provider more about your surgery?							
No	115	0.1 (< 0.1–0.3)	0.07	2.9 (1.5–6.5)	0.06	2.3 (1.6–5.0)	0.28
Yes	25	0.2 (< 0.1–0.5)		4.0 (2.6–11.5)		3.3 (1.8–6.1)	
Missing^b^	40						
Did using the SURPAS tool and seeing the personal risks affect your decision to have or not have the operation?							
No	133	0.1 (< 0.1–0.3)	0.30	3.3 (1.6–8.2)	0.40	2.6 (1.7–5.5)	0.26
Yes	27	0.1 (0.1–0.3)		3.9 (1.9–8.9)		3.3 (1.9–5.5)	
Missing^b^	20						

^a^*Abbreviations*: *IQR* interquartile range, *SURPAS* Surgical Risk Preoperative Assessment System

^b^Missing was due to no responses to the survey for the given question

†Since the risk estimates were skewed, the *P* values were calculated by Wilcoxon rank sum and bolded when less than 0.05

#### Relationship of the providers’ survey answers to the patients’ risk estimates

We investigated if there were any relationships between patient risk estimates and how surgeons responded to those same four questions (Table 4). Patient risk had a profound effect on the providers’ responses: as patient risk increased, providers had less certainty about their expectation of the patient’s risk compared to SURPAS calculations of that risk; they perceived SURPAS as being more helpful; and they thought that the SURPAS tool changed their interaction with the patient (all *p*-values < 0.0001).

**Table 4:** Median and interquartile range of risk estimates stratified by response to surgeon survey question related to the SURPAS tool

Question^a^		30-day mortality		Overall morbidity		Unplanned Readmission	
	N	Median (IQR)	*P* value†	Median (IQR)	*P* value†	Median (IQR)	*P* value†
Was the surgical risk provided from SURPAS:							
The same	94	< 0.1 (< 0.1–0.2)	**< 0.0001**	1.8 (1.3–4.9)	**< 0.0001**	1.9 (1.3–3.3)	**< 0.0001**
Less	26	0.2 (0.1–0.5)		6.3 (3.2–14.9)		4.5 (3.0–8.6)	
Greater than you expected it to be	36	0.7 (0.2–1.9)		10.7 (5.3–14.4)		5.9 (3.7–7.4)	
Missing‡	24						
On a scale of 1–4, please circle the number that best reflects your opinion of the risk document given to the patient?							
It was not helpful	18	< 0.1 (< 0.1- < 0.1)	**< 0.0001**	1.6 (1.0–1.8)	**< 0.0001**	1.8 (1.2–1.9)	**< 0.0001**
It was somewhat helpful	44	0.1 (< 0.1–0.3)		2.8 (1.5–5.8)		2.3 (1.4–4.4)	
It was helpful	91	0.1 (< 0.1–0.5)		4.1 (1.7–11.2)		3.3 (1.9–5.9)	
It was very helpful	20	0.4 (0.1–2.0)		11.9 (3.9–22.4)		5.6 (3.7–9.8)	
Missing^b^	7						
Did using the SURPAS tool change the interaction with the patient during the preoperative clinic visit?							
No	87	< 0.1 (< 0.1–0.2)	**< 0.0001**	1.9 (1.4–5.3)	**< 0.0001**	1.9 (1.43–3.7)	**< 0.0001**
Yes	85	0.2 (< 0.1—1.1)		5.5 (2.8–12.3)		4.0 (2.3–6.5)	
Missing^b^	8						
If yes: Did you find the change to be:							
Detrimental	0	0 (0–0)	0.72	0 (0–0)	0.33	0 (0–0)	0.37
Neutral	4	0.1 (0.1–1.4)		3.1 (1.3–9.9)		2.8 (1.3–5.3)	
Beneficial	80	0.2 (< 0.1–1.1)		5.5 (2.9–12.6)		4.1 (2.3–6.7)	
Missing^b^	1						

^a^*Abbreviations*: *IQR* interquartile range, *SURPAS* Surgical Risk Preoperative Assessment System

^b^Missing was due to no responses to the survey for the given question

†Since the risk estimates were skewed, the *P* values were calculated by Wilcoxon rank sum and bolded when less than 0.05

## Discussion

The objective of an informed decision is for the patients to understand the relevant risks and benefits of treatment so that they can more effectively incorporate their own values and perspective along with the provider’s into a mutually agreed upon choice of care [25]. The use of patient’s individualized risk assessment during the preoperative evaluation has the potential to improve the process of informed consent during the preoperative patient consultation [13]. We performed a trial implementation of SURPAS in elective surgical patients across a broad range of surgical clinics and assessed patient and provider perceptions of SURPAS as a risk assessment tool utilizing a mixed methodology to inform the subsequent development of SURPAS.

In this study, the majority of patients reported that they had a discussion of surgical risk during the preoperative consultation using the SURPAS document displaying the patient’s risks. Most patients reported that they understood their surgical risk very well following their discussion of surgical risk. This suggests that SURPAS improves patient understanding of their individual risks of postoperative complications. All of the patients who received the SURPAS document found it to be helpful with most finding it very helpful as a risk assessment tool. The majority of patients we interviewed liked the handout, which explained and compared individual risk to the national average. The handout especially comforted them when their risk was low. They also shared that the handout was helpful to explain the risk of surgery to their family members and that they didn’t need to focus on remembering the risk discussion because risk results were written down.

Patients who had higher risk estimates were more likely to desire increased discussion of surgical risk with their surgeon. On the other hand, patient risk estimates appeared unrelated to the likelihood of the patient changing their decision to undergo the operation. Thus, while SURPAS has the potential to influence patient’s decisions, in this patient cohort it did not influence their decision to undergo surgery.

When patient risk estimates were low surgeons were more likely to report that the risk estimates provided by SURPAS were the same as they expected. However, when patient risk estimates were slightly higher than nominal, surgeons were more likely to overestimate patient risk and report that the SURPAS estimates were less than expected. When patient risk estimates were much higher than nominal, they were more likely to underestimate patient risk and report that the SURPAS estimates were greater than expected. This suggests that surgeons are fairly good at determining which patients are low risks for the outcomes evaluated but they are less precise at approximating patient risk when it is moderate to severe. Furthermore, as patient risk estimates increased, surgeons increasingly found the SURPAS handout to be more helpful as a risk assessment tool. Finally, surgeons were more likely to change their interaction with the patient as risk estimates increased, with the majority reporting that the change was beneficial. This further supports the usefulness of SURPAS as a risk assessment tool.

There are several strengths and limitations of this study. Strengths include the mixed methods approach, combining qualitative and quantitative data; and the examination of the relationships between the level of patient risk and the patients’ and providers’ perceptions of the tool. The primary limitations of the study were a limited sample size of patients and providers from one center, the slightly lower risk of the patients compared to the national ACS NSQIP database, and some survey data not being reported by patients and providers.

The next step in SURPAS development will be the widespread dissemination and implementation of SURPAS into surgical programs throughout the University of Colorado Health System and other interested health systems throughout the country. The overall approach to understand how to best implement and disseminate SURPAS is grounded in Rogers’s Diffusion of Innovation theory [26]. The theory posits that within an organization, information gathering, and planning precedes the implementation of the innovation. Adapting and integrating these theoretical components allow us to better understand the SURPAS process to reduce surgical complications by highlighting the importance of specific contextual components, personnel abilities and opinions, technological capabilities, and patients’ opinions and satisfaction with processes, which will ultimately impact the decision to adopt and implement the SURPAS innovation [27].

## Conclusions

Patients and providers were supportive of the use of SURPAS in the preoperative risk assessment of the patients, thus improving the surgical risk assessment process. Patients thought that SURPAS was helpful regardless of what their risk level was, whereas providers thought that SURPAS was more helpful in the higher risk patients. However, surgeons were not as accurate at predicting patient risk once it rose above nominal rates. Overall, SURPAS proved helpful to patients and providers in engaging in the risk assessment discussion around the decision for surgery.

## Supplementary information

**Additional file 1: eTable 1.** Demographics of patient undergoing elective surgery in the University of Colorado Hospital National Surgical Quality Improvement Program from January 1, 2017 to July 31, 2017.

**Additional file 2: eTable 2.** List of procedure types undergone by study patients.

## Data Availability

The datasets used and/or analyzed during the current study are available from the corresponding author on reasonable request.

## Abbreviations

ACS: American College of Surgeons
ASA class: American Society of Anesthesiology physical status classification
HER: Electronic health record
IQR: Interquartile range
NP: Nurse practitioner
SD: Standard Deviation
SURPAS: Surgical Risk Preoperative Assessment System
