# Supplementary material for: The value of the “Surgical Risk Preoperative Assessment System” (SURPAS) in preoperative consultation for elective surgery: a pilot study
Source: Patient Saf Surg. 2020 Jul 25;14:31. doi: 10.1186/s13037-020-00256-4 (PMC7382083; doi:10.1186/s13037-020-00256-4)
Supplement: Supplementary file 1 — Additional file 1: eTable 1. Demographics of patient undergoing elective surgery in the University of Colorado Hospital National Surgical Quality Improvement Program from January 1, 2017 to July 31, 2017. [file 13037_2020_256_MOESM1_ESM.docx]

eTable 1. Demographics of patient undergoing elective surgery in the University of Colorado Hospital National Surgical Quality Improvement Program from January 1, 2017 to July 31, 2017.

| Characteristics | | Cohort  (n=1,341)  N (%) |
| --- | --- | --- |
| Age, years, mean (SD) | | 52.6 (16.6) |
| Gender | |  |
|  | Female | 745 (55.6) |
|  | Male | 596 (44.4) |
| Race/ethnicity | |  |
|  | White, not of Hispanic origin | 944 (70.4) |
|  | Hispanic origin | 158 (11.8) |
|  | Black, not of Hispanic origin | 104 (7.8) |
|  | Asian or Pacific Islander | 33 (2.5) |
|  | American Indian or Alaska Native | 5 (0.4) |
|  | Unknown | 97 (7.2) |
