# Supplementary material for: The value of the “Surgical Risk Preoperative Assessment System” (SURPAS) in preoperative consultation for elective surgery: a pilot study
Source: Patient Saf Surg. 2020 Jul 25;14:31. doi: 10.1186/s13037-020-00256-4 (PMC7382083; doi:10.1186/s13037-020-00256-4)
Supplement: Supplementary file 2 — Additional file 2: eTable 2. List of procedure types undergone by study patients. [file 13037_2020_256_MOESM2_ESM.docx]

| Description | Frequency | Percent | Cumulative Frequency | Cumulative Percent |
| --- | --- | --- | --- | --- |
| Parathyroidectomy or exploration of parathyroid(s); | 26 | 13.20 | 26 | 13.20 |
| Missing | 17 | 8.63 | 43 | 21.83 |
| Thyroidectomy, total or complete | 16 | 8.12 | 59 | 29.95 |
| Total thyroid lobectomy, unilateral; with or without isthmusectomy | 11 | 5.58 | 70 | 35.53 |
| Reconstructive repair of pectus excavatum or carinatum; minimally invasive approach (Nuss procedure), with thoracoscopy | 8 | 4.06 | 78 | 39.59 |
| Total thyroid lobectomy, unilateral; with contralateral subtotal lobectomy, including isthmusectomy | 8 | 4.06 | 86 | 43.65 |
| Thoracoscopy, surgical; with lobectomy (single lobe) | 6 | 3.05 | 92 | 46.70 |
| Thoracoscopy, surgical; with therapeutic wedge resection (eg, mass, nodule), initial unilateral | 6 | 3.05 | 98 | 49.75 |
| Cystectomy, complete, with ureteroileal conduit or sigmoid bladder, including intestine anastomosis; with bilateral pelvic lymphadenectomy, including external iliac, hypogastric, and obturator nodes | 3 | 1.52 | 101 | 51.27 |
| Imbrication of diaphragm for eventration, transthoracic or transabdominal, paralytic or nonparalytic | 3 | 1.52 | 104 | 52.79 |
| Thoracoscopy, surgical; with excision of mediastinal cyst, tumor, or mass | 3 | 1.52 | 107 | 54.31 |
| Thoracoscopy, surgical; with thoracic sympathectomy | 3 | 1.52 | 110 | 55.84 |
| Arteriovenous anastomosis, open; by upper arm cephalic vein transposition | 2 | 1.02 | 112 | 56.85 |
| Arthroplasty, acetabular and proximal femoral prosthetic replacement (total hip arthroplasty), with or without autograft or allograft | 2 | 1.02 | 114 | 57.87 |
| Arthroplasty, glenohumeral joint; total shoulder (glenoid and proximal humeral replacement (eg, total shoulder)) | 2 | 1.02 | 116 | 58.88 |
| Cervical lymphadenectomy (modified radical neck dissection) | 2 | 1.02 | 118 | 59.90 |
| Cystourethroscopy with ureteroscopy; with treatment of ureteral stricture (eg, balloon dilation, laser, electrocautery, and incision) | 2 | 1.02 | 120 | 60.91 |
| Endovascular repair of infrarenal abdominal aortic aneurysm or dissection; using modular bifurcated prosthesis (2 docking limbs) | 2 | 1.02 | 122 | 61.93 |
| Esophagogastroduodenoscopy, flexible, transoral; with transendoscopic ultrasound-guided intramural or transmural fine needle aspiration/biopsy(s), (includes endoscopic ultrasound examination limited to the esophagus, stomach or duodenum, and adjacent structures) | 2 | 1.02 | 124 | 62.94 |
| Excision first and/or cervical rib; | 2 | 1.02 | 126 | 63.96 |
| Excision of chest wall tumor involving ribs, with plastic reconstruction; without mediastinal lymphadenectomy | 2 | 1.02 | 128 | 64.97 |
| Excision, tumor, soft tissue of neck or anterior thorax, subcutaneous; less than 3 cm | 2 | 1.02 | 130 | 65.99 |
| Laparoscopy, surgical, with adrenalectomy, partial or complete, or exploration of adrenal gland with or without biopsy, transabdominal, lumbar or dorsal | 2 | 1.02 | 132 | 67.01 |
| Mediastinoscopy, includes biopsy(ies), when performed | 2 | 1.02 | 134 | 68.02 |
| Mediastinotomy with exploration, drainage, removal of foreign body, or biopsy; transthoracic approach, including either transthoracic or median sternotomy | 2 | 1.02 | 136 | 69.04 |
| Parathyroid autotransplantation (List separately in addition to code for primary procedure) | 2 | 1.02 | 138 | 70.05 |
| Thoracoscopy, surgical; with pleurodesis (eg, mechanical or chemical) | 2 | 1.02 | 140 | 71.07 |
| Thoracoscopy; with diagnostic biopsy(ies) of lung nodule(s) or mass(es) (eg, wedge, incisional), unilateral | 2 | 1.02 | 142 | 72.08 |
| Thymectomy, partial or total; sternal split or transthoracic approach, without radical mediastinal dissection (separate procedure) | 2 | 1.02 | 144 | 73.10 |
| Thyroidectomy, total or subtotal for malignancy; with limited neck dissection | 2 | 1.02 | 146 | 74.11 |
| Thyroidectomy, total or subtotal for malignancy; with radical neck dissection | 2 | 1.02 | 148 | 75.13 |
| Transurethral resection of bladder neck (separate procedure) | 2 | 1.02 | 150 | 76.14 |
| Arteriovenous anastomosis, open; direct, any site (eg, Cimino type) (separate procedure) | 1 | 0.51 | 151 | 76.65 |
| Bronchoscopy, rigid or flexible, including fluoroscopic guidance, when performed; with transbronchial needle aspiration biopsy(s), each additional lobe (List separately in addition to code for primary procedure) | 1 | 0.51 | 152 | 77.16 |
| Bypass graft, with other than vein; iliofemoral | 1 | 0.51 | 153 | 77.66 |
| Cervical lymphadenectomy (complete) | 1 | 0.51 | 154 | 78.17 |
| Colonoscopy, flexible; with transendoscopic balloon dilation | 1 | 0.51 | 155 | 78.68 |
| Craniofacial approach to anterior cranial fossa; extradural, including lateral rhinotomy, ethmoidectomy, sphenoidectomy, without maxillectomy or orbital exenteration | 1 | 0.51 | 156 | 79.19 |
| Cystourethroscopy, with fulguration (including cryosurgery or laser surgery) and/or resection of; LARGE bladder tumor(s) | 1 | 0.51 | 157 | 79.70 |
| Cystourethroscopy, with fulguration (including cryosurgery or laser surgery) and/or resection of; MEDIUM bladder tumor(s) (2.0 to 5.0 cm) | 1 | 0.51 | 158 | 80.20 |
| Cystourethroscopy, with fulguration (including cryosurgery or laser surgery) and/or resection of; SMALL bladder tumor(s) (0.5 up to 2.0 cm) | 1 | 0.51 | 159 | 80.71 |
| Endovascular repair of iliac artery (eg, aneurysm, pseudoaneurysm, arteriovenous malformation, trauma) using ilio-iliac tube endoprosthesis | 1 | 0.51 | 160 | 81.22 |
| Excision of infected graft; extremity | 1 | 0.51 | 161 | 81.73 |
| Excision, tumor, soft tissue of upper arm or elbow area, subcutaneous; less than 3 cm | 1 | 0.51 | 162 | 82.23 |
| Hepatectomy, resection of liver; partial lobectomy | 1 | 0.51 | 163 | 82.74 |
| Incision and drainage, complex, postoperative wound infection | 1 | 0.51 | 164 | 83.25 |
| Laparoscopy, surgical, esophagogastric fundoplasty (eg, Nissen, Toupet procedures) | 1 | 0.51 | 165 | 83.76 |
| Laparoscopy, surgical, esophagomyotomy (Heller type), with fundoplasty, when performed | 1 | 0.51 | 166 | 84.26 |
| Laparoscopy, surgical; cholecystectomy | 1 | 0.51 | 167 | 84.77 |
| Laparoscopy, surgical; cholecystectomy with cholangiography | 1 | 0.51 | 168 | 85.28 |
| Laparoscopy, surgical; colectomy, partial, with anastomosis | 1 | 0.51 | 169 | 85.79 |
| Laparoscopy, surgical; radical nephrectomy (includes removal of Gerota's fascia and surrounding fatty tissue, removal of regional lymph nodes, and adrenalectomy) | 1 | 0.51 | 170 | 86.29 |
| Lengthening or shortening of tendon, leg or ankle; multiple tendons (through same incision), each | 1 | 0.51 | 171 | 86.80 |
| Ligation, division, and stripping, short saphenous vein | 1 | 0.51 | 172 | 87.31 |
| Mastectomy, partial (eg, lumpectomy, tylectomy, quadrantectomy, segmentectomy); | 1 | 0.51 | 173 | 87.82 |
| Nephrectomy, partial | 1 | 0.51 | 174 | 88.32 |
| Pancreatectomy, proximal subtotal with total duodenectomy, partial gastrectomy, choledochoenterostomy and gastrojejunostomy (Whipple-type procedure); with pancreatojejunostomy | 1 | 0.51 | 175 | 88.83 |
| Pancreatectomy, proximal subtotal with total duodenectomy, partial gastrectomy, choledochoenterostomy and gastrojejunostomy (Whipple-type procedure); without pancreatojejunostomy | 1 | 0.51 | 176 | 89.34 |
| Recipient nephrectomy (separate procedure) | 1 | 0.51 | 177 | 89.85 |
| Removal of lung, other than pneumonectomy; single lobe (lobectomy) | 1 | 0.51 | 178 | 90.36 |
| Repair initial inguinal hernia, age 5 years or older; reducible | 1 | 0.51 | 179 | 90.86 |
| Repair of nasal vestibular stenosis (eg, spreader grafting, lateral nasal wall reconstruction) | 1 | 0.51 | 180 | 91.37 |
| Repair umbilical hernia, age 5 years or older; reducible | 1 | 0.51 | 181 | 91.88 |
| Resection of mediastinal tumor | 1 | 0.51 | 182 | 92.39 |
| Revascularization, endovascular, open or percutaneous, femoral, popliteal artery(s), unilateral; with transluminal stent placement(s) and atherectomy, includes angioplasty within the same vessel, when performed | 1 | 0.51 | 183 | 92.89 |
| Revascularization, endovascular, open or percutaneous, tibial, peroneal artery, unilateral, initial vessel; with transluminal angioplasty | 1 | 0.51 | 184 | 93.40 |
| Revision of total hip arthroplasty; acetabular component only, with or without autograft or allograft | 1 | 0.51 | 185 | 93.91 |
| Stab phlebectomy of varicose veins, 1 extremity; 10-20 stab incisions | 1 | 0.51 | 186 | 94.42 |
| Stab phlebectomy of varicose veins, 1 extremity; more than 20 incisions | 1 | 0.51 | 187 | 94.92 |
| Thoracoscopy, surgical; with resection of thymus, unilateral or bilateral | 1 | 0.51 | 188 | 95.43 |
| Thoracoscopy; with diagnostic biopsy(ies) of lung infiltrate(s) (eg, wedge, incisional), unilateral | 1 | 0.51 | 189 | 95.94 |
| Thoracotomy; with open intrapleural pneumonolysis | 1 | 0.51 | 190 | 96.45 |
| Thromboendarterectomy, including patch graft, if performed; carotid, vertebral, subclavian, by neck incision | 1 | 0.51 | 191 | 96.95 |
| Thymectomy, partial or total; sternal split or transthoracic approach, with radical mediastinal dissection (separate procedure) | 1 | 0.51 | 192 | 97.46 |
| Thyroidectomy, including substernal thyroid; sternal split or transthoracic approach | 1 | 0.51 | 193 | 97.97 |
| Tracheoplasty; intrathoracic | 1 | 0.51 | 194 | 98.48 |
| Transcatheter placement of intravascular stent(s), cervical carotid artery, open or percutaneous, including angioplasty, when performed, and radiological supervision and interpretation; without distal embolic protection | 1 | 0.51 | 195 | 98.98 |
| Transurethral electrosurgical resection of prostate, including control of postoperative bleeding, complete (vasectomy, meatotomy, cystourethroscopy, urethral calibration and/or dilation, and internal urethrotomy are included) | 1 | 0.51 | 196 | 99.49 |
| Tympanoplasty without mastoidectomy (including canalplasty, atticotomy and/or middle ear surgery), initial or revision; with ossicular chain reconstruction and synthetic prosthesis (eg, partial ossicular replacement prosthesis [PORP], total ossicular replacement prosthesis [TORP]) | 1 | 0.51 | 197 | 100.00 |
